# Public Knowledge About How Common Chronic Diseases Affect Wound Healing Postoperatively in Aseer Region

**DOI:** 10.7759/cureus.29790

**Published:** 2022-09-30

**Authors:** Muneer J Bhat, Hussam Y Ayed, Ali M Alrasheed, Majdoleen A Alghamdi, Saud S Alsaleh, Yazeed M Alrashid, Saud Bin-fudhayl

**Affiliations:** 1 Anesthesiology, King Khalid University, Abha, SAU; 2 Medicine, King Khalid University, Abha, SAU

**Keywords:** saudi arabia, knowledge, population, factors affecting, healing, post-surgical wound

## Abstract

Background

Wound healing has classically been described to occur in three phases, regardless of the mechanism of injury. These phases are the inflammatory, proliferative, and remodeling phases. Chronic diseases adversely affect the wound healing process, and more needs to be done for different policies, such as adjustment of drug therapy, diet, or behavior to help rapid wound healing. Diabetes, auto-immune diseases, obesity, malnutrition, cardiovascular disease, chronic renal disease, and cancers are the frequent co-morbidities affecting wound healing.

Aim

To assess the public knowledge about how common chronic diseases affect wound healing postoperatively in Aseer Region.

Methodology

A descriptive cross-sectional study targeting the general population living in the Aseer region for at least a year. Data were collected using a pre-structured electronic questionnaire initiated by the researchers after an intense literature review and experts’ consultation. The study questionnaire covered participants’ data, smoking and medical history, surgical history, and participant knowledge items. A questionnaire was used as a digital survey and distributed to all participants in a private and anonymous process.

Results

A total of 502 participants completed the study questionnaire. Participants' ages ranged from 18 to 60 years, with a mean age of 34.6 ± 12.9 years old. A total of 294 (58.6%) participants were males, and 341 (67.9%) were university graduates. Exact 430 (85.7%) know that Supervision and control of Diabetes Mellitus help in wound healing, 369 (73.5%) reported that Chronic diseases delay wound healing, and 449 (89.4%) think that commitment to therapeutic and preventive plans before and after any surgical procedure contributing in rapid wound healing for chronic diseases patients. As for the effect of chronic diseases on a surgical wound, 320 (63.7%) reported delayed wound healing, 241 (48%) knew it may increase the infection, and 186 (37.1%) reported it might Decrease blood supply to the site of a wound.

Conclusions

In conclusion, the study revealed that more than half of the population in Aseer regions were knowledgeable regarding the effect of chronic diseases on post-optative wound healing, especially for the benefit of a commitment to therapeutic and preventive plans before and after any surgical procedure.

## Introduction

Usually, the healing process of wounds in healthy persons passes through an orderly sequence of physiologic events [1]. Numerous factors cause delayed wound healing, leading to chronic nonhealing wounds and ulcers or complicating the surgical course [2]. The risk factors associated with impaired wound healing include patient factors, underlying disease, and wound management [3].

Wound healing has classically been described to occur in 4 phases, regardless of the mechanism of injury. These phases are hemostasis, inflammatory, proliferative, and remodeling [4-6]. Wound healing includes three types which are primary, secondary, and tertiary healing. In most surgical wound tracks, a primary healing mechanism is minimal tissue loss, and the wound edges can come acceptably closer. This allows for primary healing, which is featured by rapid epithelialisation of the wound with slight scarring [7].

Chronic diseases adversely affect the wound healing process, and more needs different policies such as adjustment of drug therapy, diet, or behavior to help rapid wound healing. Diabetes, auto-immune diseases, obesity, malnutrition, cardiovascular disease, chronic renal disease, and cancers are the frequent co-morbidities affecting wound healing [8-10]. 

Population with a lack of knowledge regarding wound care and its relation to co-morbidities may cause undesirable consequences, including delayed healing, higher healthcare burden, financial impact, and reduced quality of life. So, it is vital to improving public awareness about various factors on wound healing. Minimal studies are available in the literature regarding the assessment of knowledge and attitude of patients towards caring for wound injuries [11-13]. The current study aimed to assess public knowledge about how common chronic diseases affect wound healing postoperatively in the Aseer Region, Southern Saudi Arabia.

## Materials and methods

A descriptive cross-sectional study targeted the general population living in the Aseer region for at least a year. Data were collected using a pre-structured electronic questionnaire initiated by the researchers after an intensive literature review and experts’ consultation that fulfills the purpose of the study to avoid errors in data collection. A panel of 3 experts in the field of the study issue reviewed the questionnaire to assess its clarity and content validity. The study questionnaire covered participants’ data, including age, gender, educational level, work and monthly income, smoking, and medical history. The second section included surgical history, site of surgery, type of surgery, wound healing, and complications. The third section included participants’ knowledge regarding the effect of chronic diseases on postoperative wound healing. The last section included a question focused on self-rating regarding the effect of chronic diseases on postoperative wound healing based on a 0-5 scale. A questionnaire was used as a digital survey and distributed to all participants in a private and anonymous process. The question was designed to elicit information concisely and objectively. In addition, logic was used in the question so the subsequent answer would base on the initial response. The final questionnaire was uploaded online using social media platforms.

After data were extracted, it was revised, coded, and fed to statistical software IBM SPSS version 22(SPSS, Inc. Chicago, IL). All statistical analysis was done using two-tailed tests, and a P value less than 0.05 was statistically significant. Each correct answer was scored one point for knowledge and awareness items, and the total summation of the discrete scores of the different items was calculated. A participant with less than 60% of the total score had less awareness, while satisfactory awareness was considered if they scored 60% or more. Descriptive analysis based on frequency and percent distribution was done for all variables, including participants’ data, smoking, medical history, and surgical history with wound healing information. Also, participants’ knowledge and awareness regarding the effect of chronic diseases on postoperative wound healing were described in frequency tables and graphed.

Additionally, participants’ perceptions regarding the effect of chronic diseases on wound healing postoperative were graphed. Crosstabulation was used to assess Factors associated with public knowledge regarding the effect of chronic diseases on postoperative wound healing. Relations were tested using Pearson chi-square and exact probability tests for small frequency distributions.

## Results

A total of 502 participants completed the study questionnaire. Participants ranged from 18 to 60 years, with a mean age of 34.6 ± 12.9. A total of 294 (58.6%) participants were males, 341 (67.9%) were university graduates, and 131 (26.1%) had a secondary level of education. A total of 316 (62.9%) were married, and 251 (50%) were not employed/retired, while 144 (28.7%) were employed in the government department and 65 (12.9%) were in the military department, but 42 (8.4%) were at the private department. As for chronic diseases, 79 (15.9%) were obese with a BMI of more than 30, 48 (9.6%) had cardiovascular disease, 42 (8.4%) complained of respiratory disease (COPD, Asthma, ILD), and 22 (4.4%0 had Diabetes Mellitus while 316 (62.9%) were free of chronic diseases. Smoking was reported among 81 (16.1%) participants and 78 (15.5%0 previously used cortisone therapy (Table 1).

**Table 1:** Bio-demographic data of study participants, Aseer region, Saudi Arabia SR- Saudi Riyal; CVD- Cardiovascular disease; HTN- Hypertension; DM- Diabetes mellitus

Bio-demographic data	No	%
Age in years		
< 25	153	30.5%
25-45	193	38.4%
> 45	156	31.1%
Gender		
Male	294	58.6%
Female	208	41.4%
Educational level		
Below secondary	30	6.0%
Secondary	131	26.1%
University / above	341	67.9%
Marital status		
Single	166	33.1%
Married	316	62.9%
Divorced / widow	20	4.0%
Work sector		
Not working/retired	251	50.0%
Governmental sector	144	28.7%
Private sector	42	8.4%
Military sector	65	12.9%
Monthly income		
< 5000 SR	187	37.3%
5000-15000 SR	205	40.8%
> 15000 SR	110	21.9%
Chronic diseases		
None	316	62.9%
DM	22	4.4%
CVD & HTN	48	9.6%
Autoimmune diseases	12	2.4%
Obesity	79	15.7%
Respiratory diseases	42	8.4%
Chronic renal diseases	11	2.2%
Others	17	3.4%
Smoking		
Yes	81	16.1%
No	421	83.9%
Used cortisone therapy		
Yes	78	15.5%
No	424	84.5%

Table 2 represents surgery history among study participants in the Aseer region, Saudi Arabia. The exact 270 (53.8%) participants underwent surgery which was on the chest and abdomen among 146 (54.1%), on the lower half of the body among 54 (20%), and on the head among 35 (13%). It was open surgery among 14 (5.2%), while 256 (94.8%) had undergone closed surgery (laparoscopy, ENT surgery such as tonsillectomy or bleeding). A total of 97 (35.9%) reported that they needed 1-2 weeks for complete wound healing, 72 (26.7%) needed 2-4 weeks, and 33 (12.2%) needed more than one month. Exact of 55 (20.4%) experienced wound inflammation, 32 (11.9%) had wound infection with pus or abscess, and bleeding was reported among 25 (9.3%), while 186 (68.9%) had no complications.

**Table 2:** Surgery history among study participants, Aseer region, Saudi Arabia

Surgery history	No	%
Previously undergone surgery		
Yes	270	53.8%
No	232	46.2%
Site of surgery (n=270)		
Head	35	13.0%
Upper & lower limbs	23	8.5%
Chest & abdomen	146	54.1%
The back	12	4.4%
Lower half of the body	54	20.0%
Type of surgery (n=270)		
Open surgery	14	5.2%
Closed surgery	256	94.8%
Duration till wound healing (n=270)		
1-3 days	28	10.4%
3-7 days	40	14.8%
1-2 weeks	97	35.9%
2-4 weeks	72	26.7%
> 1 month	33	12.2%
Wound complications (n=270)		
Inflammation	55	20.4%
Infection	32	11.9%
Bleeding	25	9.3%
None	186	68.9%

Table 3 reveals public knowledge about how common chronic diseases affect wound healing postoperatively in Aseer Region, Saudi Arabia. Exact 430 (85.7%) know that Supervision and control of DM help in wound healing, 369 (73.5%) reported that Chronic diseases delay wound healing, and 449 (89.4%) think that commitment to therapeutic and preventive plans before and after any surgical procedure contributing in rapid wound healing for chronic diseases patients. As for the effect of chronic diseases on a surgical wound, 320 (63.7%) reported it delays wound healing, 241 (48%) know it may increase the infection, and 186 (37.1%) reported it might Decrease blood supply to the site of a wound.

**Table 3:** Public knowledge about how common chronic diseases affecting wound healing postoperatively in Aseer Region, Saudi Arabia

Knowledge items	No	%
Supervision and control of DM help in wound healing		
Yes	430	85.7%
No	18	3.6%
Don't know	54	10.8%
Chronic diseases delay wound healing		
Yes	369	73.5%
No	51	10.2%
Don't know	82	16.3%
Commitment to therapeutic and preventive plan before and after any surgical procedure contributes to rapid wound healing for chronic disease patients?		
Yes	449	89.4%
No	12	2.4%
Don't know	41	8.2%
Effect of chronic diseases on surgical wound		
Delayed healing	320	63.7%
Increase risk of infection	241	48.0%
Decrease blood supply to the site of the wound	186	37.1%
No effect	5	1.0%
Don't know	56	11.2%

**Figure 1:**
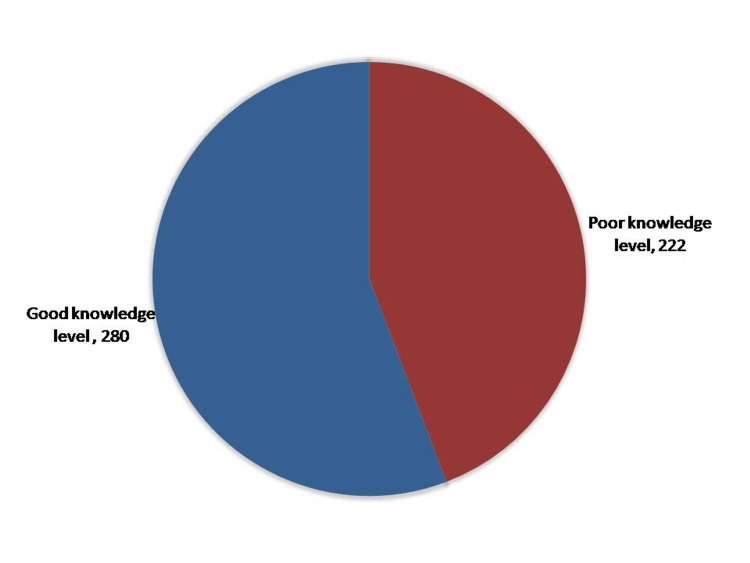
Overall Public knowledge about how common chronic diseases affect wound healing postoperatively

Figure 1 reveals the overall Public knowledge about how common chronic diseases affect wound healing postoperatively. A total of 280 (55.8%) had good knowledge of chronic disease effects, while 222 (44.2%) had poor knowledge.

**Figure 2:**
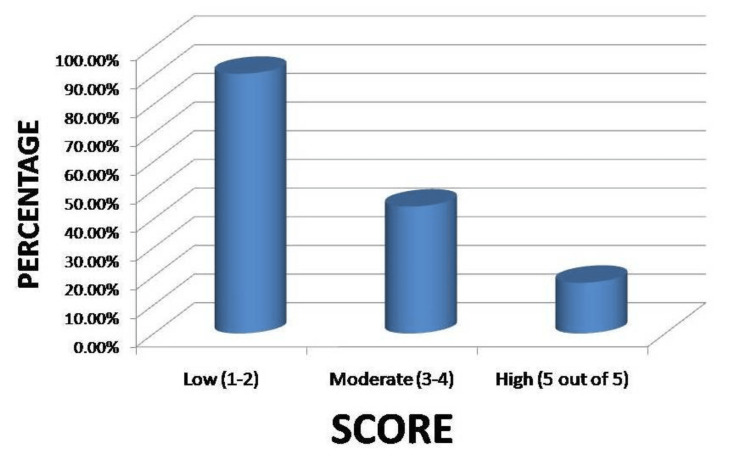
Participants’ perception regarding the dangerous effect of chronic diseases on wound healing postoperative.

Figure 2 shows participants’ perceptions regarding the dangerous effects of chronic diseases on wound healing postoperative. A total of 64 (12.7%) reported diseases had a low effect on postoperative wound healing (1-2 out of 5), and 325 (64.7%) think chronic diseases had a moderate effect. In comparison, 113 (22.5%) think it greatly affects wound healing.

Table 4 shows the factors associated with public knowledge regarding the effect of chronic diseases on postoperative wound healing. Good knowledge was detected among 59.2% of male participants compared to 51% of females with recorded statistical significance (P=.049). Also, 61.9% of university graduates had good knowledge levels versus 33.3% of others with below secondary level of education (P=0.001). Additionally, 67.3% of participants with a monthly income exceeding 15000 SR had a good knowledge level compared to 49.2% of others with low income (P=.010). 82.3% of participants who think that chronic diseases had a high effect on wound healing had good knowledge compared to others who think it had a low effect (P=0.001).

**Table 4:** Factors associated with public knowledge regarding the effect of chronic diseases on post-operative wound healing. P: Pearson X^2 ^test; $: Exact probability test * P: 0.05 (significant)

Factors		Knowledge level				p-value
		Poor		Good		
		No	%	No	%	
Age in years	< 25	66	43.10%	87	56.90%	0.884
	26-45	88	45.60%	105	54.40%	
	> 45	68	43.60%	88	56.40%	
Gender	Male	120	40.80%	174	59.20%	.049*
	Female	102	49.00%	106	51.00%	
Educational level	Below secondary	20	66.70%	10	33.30%	.001*
	Secondary	72	55.00%	59	45.00%	
	University / above	130	38.10%	211	61.90%	
Marital status	Single	69	41.60%	97	58.40%	.137^$^
	Married	140	44.30%	176	55.70%	
	Divorced / widow	13	65.00%	7	35.00%	
Monthly income	< 5000 SR	95	50.80%	92	49.20%	.010*
	5000-15000 SR	91	44.40%	114	55.60%	
	> 15000 SR	36	32.70%	74	67.30%	
Smoking	Yes	42	51.90%	39	48.10%	0.131
	No	180	42.80%	241	57.20%	
Used cortisone therapy	Yes	31	39.70%	47	60.30%	0.386
	No	191	45.00%	233	55.00%	
Previously undergone surgery	Yes	122	45.20%	148	54.80%	0.64
	No	100	43.10%	132	56.90%	
Type of surgery	Open surgery	8	57.10%	6	42.90%	0.356
	Closed surgery	114	44.50%	142	55.50%	
Wound complications	Yes	36	42.90%	48	57.10%	0.605
	No	86	46.20%	100	53.80%	
Duration till wound healing	< 2 weeks	72	43.60%	93	56.40%	0.521
	> 2 weeks	50	47.60%	55	52.40%	
Chronic diseases	Yes	91	47.90%	99	52.10%	0.196
	No	131	42.00%	181	58.00%	
Rate how dangerous effect of chronic diseases on wound healing postoperative are	Low (1-2)	58	90.60%	6	9.40%	.001*
	Moderate (3-4)	144	44.30%	181	55.70%	
	High (5 out of 5)	20	17.70%	93	82.30%	

## Discussion

Wound healing is a complex process that passes through many stages that represent the final result of multifaceted biochemical and cellular actions [14]. The current study aimed to assess public knowledge about how common chronic diseases affect wound healing postoperatively in the Aseer Region, Southern Saudi Arabia.

The study results showed that about one-third of the participants had chronic health problems, mainly cardiovascular and respiratory diseases. Also, more than half of the study respondents had undergone surgery which was mainly chest and abdominal surgeries. Wound healing duration exceeding two weeks was reported among more than one-third of them, as one-fifth experienced postoperative wound inflammation, and only 10% had post-surgical wound infection. Literature showed that infection was the most reported complication, and patients with vascular diseases are at risk of this due to poor blood flow and a wound [15].

As a result of local variations in comorbid conditions and their treatment, it is vital to study the demographics of the patients with chronic diseases that may influence the healing and persistence of the postoperative wound. Obesity and the associated co-morbidities are crucial in management and therapeutic purposes [16]. There are many other factors, including patients’ factors like age, Body mass index(BMI), co-morbidities, medications, smoking, alcohol abuse, nonsteroidal anti-inflammatory drugs(NSAIDs), and nutritional status [17]. Systemic diseases impair wound healing, including diabetes mellitus rheumatoid arthritis and its treatment: use of steroids, disease-modifying anti-rheumatic drug (DMARD), and biological therapy, thyroxine hormone substitution [18-21].

Regarding participants’ awareness of the effect of chronic diseases on wound healing, the study revealed that more than half of them (55.8%) had satisfactory awareness levels. In more detail, the vast majority of the study participants (85.7%) know that Supervision and control of Diabetes Mellitus help in wound healing and that commitment to therapeutic and preventive plans before and after any surgical procedure contributes to rapid wound healing for chronic disease patients. About three-quarters (73.5%) told those Chronic diseases delay wound healing, and two-thirds were aware that it delays wound healing, but less than half of them (48%) know it may increase the infection, and one-third (37.1%) reported it might Decrease blood supply to the site of a wound. The best awareness was reported among male participants with high educational levels and income. Jan M et al. [22] conducted a study in the Aseer region. They found that about 29% of participants had previous experience with the surgical wound, with the most common wound site being the abdomen. The most common symptom in around 58% of participants was excessive bleeding. Participants commonly used the use of Alcohol swabs and dry gauze as wound care measures. Another study by Malaekah HM et al. [23] showed that most (71.7%) participants had good knowledge of wound care. Most participants reported medical information from nonmedical resources- from social media (42.8%) and from relatives and friends (40.6%). A study among health care workers revealed that more than half of the participants (55%) knew the definition of surgical site infections(SSI). Only one-quarter (25.2%) knew about the incidence of SSI. Geers NC et al. [24] conducted a systematic review and reported that five studies labeled systemic interventions. Four of the five studies revealed significantly improved wound healing for the intervention group. Levandovski et al. [25] suggest that higher anxiety level was significantly associated with more surgical site infections (SSI). Also, the authors found that the anxiolytic drug diazepam use may decrease the risk of infection. Flores et al. [26] state that mild perioperative hypothermia is associated with surgical wound infection, and its prevention is defensible. Furthermore, a systematic review that assessed the impact of personal and cultural views on medication adherence of patients with chronic illnesses demonstrated a significant relationship between illness perceptions and other beliefs and medication adherence [27].

## Conclusions

The study revealed that more than half of the population in Aseer regions were knowledgeable regarding the effect of chronic diseases on postoperative wound healing, especially for the benefit of a commitment to therapeutic and preventive plans before and after any surgical procedure. Higher knowledge was detected among higher education and male participants with high-income levels. Improving public awareness regarding the effect of chronic diseases on postoperative wound healing may help contribute to disease control, with decreased post-surgical wound infection and associated complications with a lower social and economic burden.
